# JWA loss promotes cell migration and cytoskeletal rearrangement by affecting HER2 expression and identifies a high-risk subgroup of HER2-positive gastric carcinoma patients

**DOI:** 10.18632/oncotarget.9211

**Published:** 2016-05-06

**Authors:** Jing Qian, Weiyou Zhu, Keming Wang, Lin Ma, Jin Xu, Tongpeng Xu, Oluf Dimitri Røe, Aiping Li, Jianwei Zhou, Yongqian Shu

**Affiliations:** ^1^ Department of Oncology, The First Affiliated Hospital of Nanjing Medical University, Nanjing, China; ^2^ Department of Oncology, The Secondary Affiliated Hospital of Nanjing Medical University, Nanjing, China; ^3^ Department of Molecular Cell Biology and Toxicology, Jiangsu Key Laboratory of Cancer Biomarkers, Prevention & Treatment, Cancer Center, Nanjing Medical University, Nanjing, China; ^4^ Department of Cancer Research and Molecular Medicine, Norwegian University of Science and Technology (NTNU), Trondheim, Norway; ^5^ Department of Clinical Medicine, Clinical Cancer Research Center, Aalborg University Hospital, Aalborg, Denmark

**Keywords:** gastric cancer, HER2, cell migration, JWA, PEA3

## Abstract

**Background and Aims:**

JWA, a microtubule-associated protein (MAP) involved in apoptosis, has been identified as a suppressor of metastasis, and it affects cell migration in melanoma and its downregulation in tumor is an idependent negative prognostic factor in resectable gastric cancer. HER2 overexpression has been observed in gastric cancer (GC) cells and implicated in the metastatic phenotype. However, the biological role of JWA in migration and its clinical value in HER2-positive GC remain elusive.

**Results:**

JWA suppresses EGF-induced cell migration and actin cytoskeletal rearrangement by abrogating HER2 expression and downstream PI3K/AKT signaling in HER2-overexpressing GC cell lines. The modulation of HER2 by JWA is dependent on ERK activation and consequent PEA3 upregulation and activation. Reduced JWA expression is associated with high HER2 expression and with poor survival in patients with AGC, whereas HER2 expression alone is not associated with survival. However, concomitant low JWA and high HER2 expression is associated with unfavorable outcomes. Additionally, when patients were stratified by JWA expression, those with higher HER2 expression in the low JWA expression subgroup exhibited worse survival.

**Methods:**

The impact of JWA on the EGF-induced migration of HER2-positive GC cells was studied using transwell assays and G-LISA assays. Western blotting, real-time PCR, electrophoretic mobility shift assays and luciferase assays were utilized to investigate the mechanisms by which JWA affects HER2. The association of JWA with HER2 and its clinical value were further analyzed by IHC in 128 pairs of advanced gastric cancer (AGC) and adjacent normal tissue samples.

**Conclusions:**

This study characterizes a novel mechanism for regulating cell motility in HER2-overexpressing GC cells involving JWA-mediated MEK/ERK/PEA3 signaling activation and HER2 downregulation. Furthermore, JWA may be a useful prognostic indicator for advanced GC and may help stratify HER2-positive patient subgroups to better identify unfavorable outcomes.

## INTRODUCTION

Gastric cancer (GC) is the third most common cause of cancer death in the world, affecting almost one million people [1]. Metastasis is the leading cause of death from gastric cancer (GC). Despite certain advances in chemotherapy regimens and targeted therapy [2–4], the 5-year survival of patients with advanced GC does not exceed 30% [5]. Deeper understanding of the mechanisms underlying metastasis would facilitate identification of predictive biomarkers and development of novel effective treatments.

Human epidermal growth factor receptor 2 (HER2/ErbB2), a member of the epidermal growth factor receptor (EGFR) family, is overexpressed in several human cancers, including 20-25% of breast cancer (BC) cases and 10-30% of GC cases [6]. HER2-positive BC is characterized by aggressiveness and high metastatic potential [7]. The HER2-directed tyrosine kinase inhibitor lapatinib and the anti-HER2 monoclonal antibody trastuzumab prolong disease-free survival and overall survival [8] as well as suppressing tumor growth and metastasis in vitro and in vivo [6]. Although the benefit of trastuzumab combined with chemotherapy was demonstrated in HER2-positive GC patients [3], the overall response rate is only approximately half of that in HER2-positive BC patients [8]. Furthermore, in contrast with the well-characterized role of HER2 in BC, the prognostic value of HER2 in GC remains elusive. These differences could be due to regulatory networks in HER2-positive GC compared with those in HER2-positive BC. Dissecting the molecular biology of metastasis in HER2-positive GC is therefore necessary to facilitate the identification of novel prognostic biomarkers and therapeutic targets for this subtype of cancer.

The JWA protein encoded by ARL6IP5, is multi-functional microtubule-associated protein (MAP) that is involved in DNA damage repair, apoptosis, and cell differentiation in various physiological contexts [9, 10]. Recent studies have revealed that JWA inhibits multiple steps of metastasis, including cell invasion, cell adhesion, and angiogenesis, in melanoma, GC and hepatocellular carcinoma [11–13]. High JWA expression has also been demonstrated to be a favorable prognostic indicator, both independently and in combination with low focal adhesion kinase (FAK) expression, in patients with resected GC [14]. Moreover, JWA is involved in cell migration in response to arsenic trioxide (As_2_O_3_) and phorbol ester (PMA) via different downstream MAPK/ERK cascades (FAK and cyclooxygenase-2 (COX-2), respectively) in cervical cancer, melanoma and hepatocellular carcinoma cells [15]. Although accumulating evidence has revealed the function of JWA in tumor metastasis, the biological role of JWA in cell migration and its clinical relevance in HER2-positive GC have not yet been explored. This study aimed to determine the impact of JWA on cell migration and the related mechanism as well as its prognostic value in HER2-positive GC.

## RESULTS

### JWA suppresses EGF-induced cell migration and cytoskeletal rearrangement

**Figure 1:**
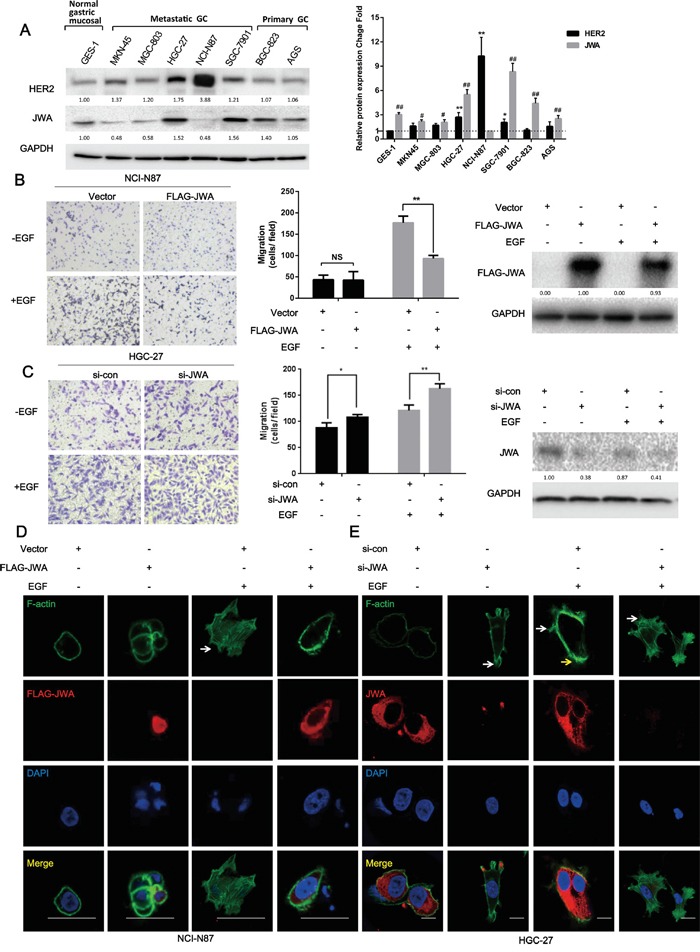
JWA inhibits cell migration and actin cytoskeletal rearrangement in HER2-overexpressing gastric cancer cells **A.** Expression of JWA and HER2 in gastric cancer (GC) cell lines and normal gastric mucosal cells. (Left panel) Equal amounts of protein from five metastatic GC cell lines (MKN-45, MGC-803, HGC-27, SGC-7901, and NCI-N87), two primary GC cell lines (BGC-823 and AGS) and normal gastric mucosal epithelial cells (GES-1) were evaluated by immunoblotting to detect JWA, HER2 and glyceraldehyde 3-phosphate dehydrogenase (GAPDH). (Right panel) The intensity of the JWA and HER2 protein bands in the 8 cell lines were analyzed by densitometry and normalized to GAPDH. The fold change in expression of each protein is expressed as a grey value ratio of each group to the control group with the lowest value among 8 cell lines. The data are plotted as the mean ± standard deviation (SD) of 3 independent replicates. Two-tailed Student's t-test; * P<0.05 and ** P<0.01 compared to the GES-1 group; # P<0.05 and ## P<0.01 compared to the NCI-N87 group. **B, C.** NCI-N87 cells overexpressing JWA or the respective vectors (B) and HGC-27 cells transfected with JWA siRNA or scramble control (C) were seeded into the upper compartments of transwells in the absence or presence of 100 ng/ml EGF for 24 h after a 12-h serum starvation. (Left panel) The panels show images at 100x of NCI-N87 and HGC-27 cells on the lower surface of the transwells. (Middle panel) The number of cells on the lower surface of the transwells was counted in five random fields. Error bars indicate the S.E.M. of three independent experiments in triplicate. Two-tailed Student's t-test; * P<0.05 and ** P<0.01. (Right panel) The expression of JWA was determined by western blot analysis. **D, E.** Phalloidin-FITC staining was performed to observe F-actin stress fibers in JWA-overexpressing NCI-N87 cells (D) and JWA-silenced HGC-27 cells with their corresponding controls (E). Scale bar, 100 μm.

Immunoblotting for JWA and HER2 in metastatic GC cell lines (MKN-45, MGC-803, HGC-27, SGC-7901, and NCI-N87), primary GC cell lines (BGC-823 and AGS) and normal gastric mucosal epithelial cells (GES-1) revealed that the NCI-N87 and HGC-27 metastatic cell lines had the highest HER2 expression among the GC cells. Thus, these two cell lines were selected as the HER2-positive cell models to explore the effects of JWA (Figure 1A).

The transwell assays showed that overexpressing JWA with the FLAG-JWA plasmid inhibited 50% of the EGF–induced motility of NCI-N87 cells, whereas there were no significant differences in the number of cells on the chamber surface between the FLAG-JWA and vector groups in the EGF-induced motility assay (Figure 1B). Likewise, siRNA targeting JWA significantly increased the migratory potential of HGC-27 cells in the presence or absence of EGF (Figure 1C).

Cell migration is a complicated process that involves the formation of cell membrane protrusions near the leading edges or microspikes, such as filopodia and lamellipodia, via the assembly of globular actin (G-actin) into fibrous actin (F-actin) [20]. Here, the F-actin distribution was analyzed by phalloidin-FITC staining, which showed fewer filopodia (white arrow) in JWA-overexpressing NCI-N87 cells treated with EGF compared with vector control cells (Figure 1D). More pronounced filopodia (white arrow) and elongated lamellipodia (yellow arrow) were observed in HGC-27 cells after JWA downregulation regardless of EGF stimulation (Figure 1E).

Although HER2 positively regulates cell proliferation and survival [21], the ectopic expression of JWA did not markedly affect the cell proliferation rate according to sensitive EdU assays (Supplementary Figure S1A, S1B), suggesting that JWA may specifically suppress GC cell motility in response to EGF.

### JWA modulates HER2 expression and downstream AKT signaling

**Figure 2:**
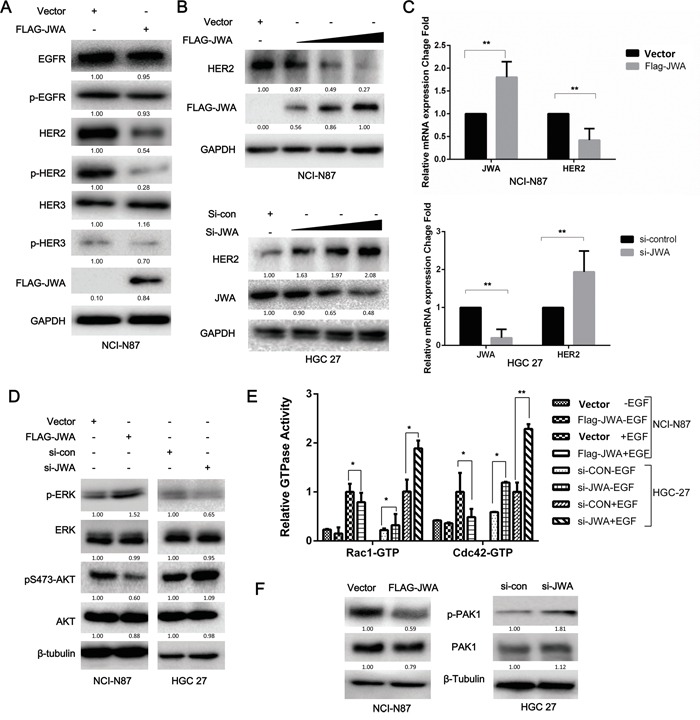
JWA represses HER2 expression and downstream AKT signaling NCI-N87 and HGC-27 cells transfected with overexpression plasmids and siRNAs were serum-starved for 12 h and then treated with EGF (100 ng/ml) for 20 min. **A.** EGFR, p-EGFR, HER2, p-HER2, HER3, and p-HER3 were examined by western blot analysis in JWA-overexpressing NCI-N87 cells. **B.** HER2 protein levels changed in a dose-dependent manner in response to JWA upregulation or downregulation. **C.** HER2 and JWA mRNA levels were detected by real-time PCR. The values were calculated as 2^−ddCT^, and the relative fold change was compared to the control groups after being normalized to GAPDH. AKT, ERK1/2 **D.**, and PAK1 **F.** were examined by western blot analysis with total and phospho-specific antibodies. **E.** The effect of JWA overexpression or silencing on the Rho GTPase guanine nucleotide-binding status of Rac1 and Cdc42 with or without EGF (100 ng/ml) was examined in cell lysates using the G-LISA assay. The data are expressed relative to the control groups (Vector NCI-87 or si-con HGC-27). The data are presented as the mean and SD of three independent experiments. * P<0.05 and ** P<0.01.

EGF-induced migratory potential is closely related to the activation of EGFR and ErbB3 by autophosphorylation, whereas HER2 is not directly activated by EGF [21]. Therefore, we investigated whether JWA influenced the expression or phosphorylation of other EGFR family members to inhibit motility. JWA reduced HER2 protein expression but did not affect the expression or activity of EGFR or ErbB3 in response to EGF stimulation (Figure 2A). Moreover, the JWA-mediated negative regulation of HER2 expression occurred in a dose-dependent manner in both NCI-N87 and HGC-27 cells after a 48-h transfection (Figure 2B). The mRNA expression of JWA and HER2 exhibited a similar trend as the protein expression (Figure 2C).

As HER2 activation induces cell migration mainly via two downstream signaling pathways, the MAPK/ERK and PI3K/Akt pathways [22], p-AKT and p-ERK levels were examined by western blotting to determine which pathway was involved in the JWA-mediated inhibition of migration. JWA activated MAPK/ERK but attenuated PI3K/Akt in NCI-N87 and HGC-27 cells (Figure 2D).

**Figure 3:**
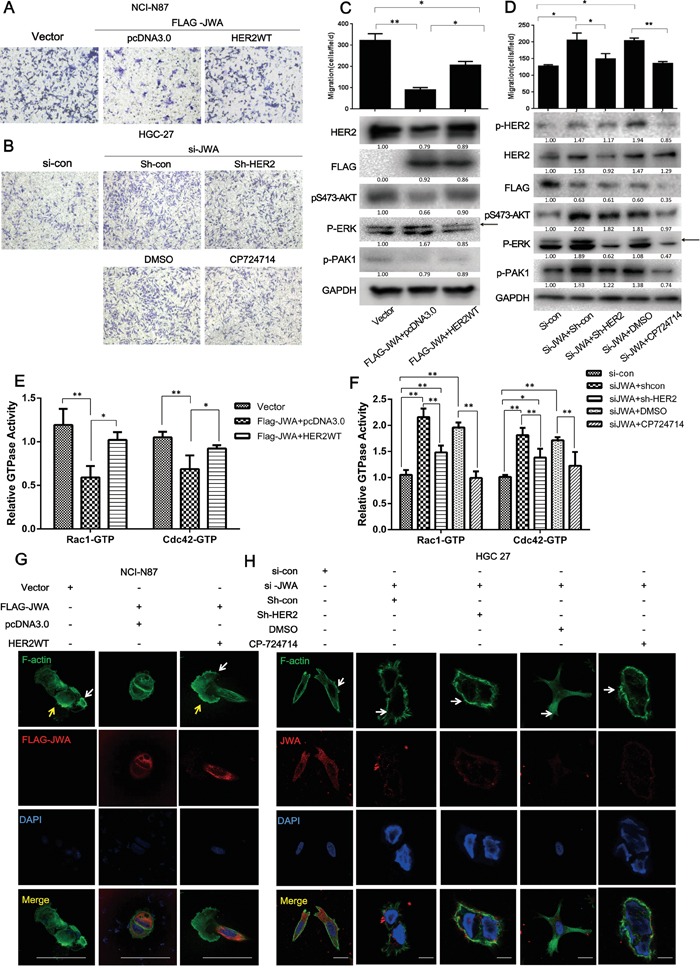
JWA inhibits cell migration by suppressing HER2 expression and downregulating downstream AKT signaling HER2 was restored by transfecting a HER2 WT plasmid into JWA-overexpressing NCI-N87 cells, and JWA-silenced HGC-27 cells were co-transfected with shHER2 for 72 h or were treated with the HER2-specific inhibitor, CP714724 (2 μM), for 48 h after incubation with JWA siRNA for 24 h. Migration assays were then performed, and microscopic images were acquired and are presented (**A, B.**, 200x magnification) with the number of cells per field (**C, D.**, upper panel). Western blots were used to analyze the protein levels of HER2, pTyr1248-HER2, p-AKT, p-PAK, FLAG-JWA, and JWA (C, D, lower panel). Arrows indicate the interested band. **E, F.** The Rho activation status of Rac1 and Cdc42 was assessed in cell lysates using the G-LISA assay, and the data are presented relative to the control groups (NCI-87 Vector or HGC-27 si-con). **G, H.** Phalloidin-FITC tracker and anti-JWA or anti-FLAG antibodies were used to stain F-actin and JWA, respectively, in the above cells for 1 h at room temperature. Fluorescent mounting media with DAPI was applied to identify the nuclei. Scale bars, 20 μm. The data are presented as the mean and SD of three independent experiments. * P<0.05; ** P<0.01; Student's t-test.

Because the PI3K/Akt signaling pathway plays a vital role in EGF-induced migration by activating Rac1 and Cdc42, that are important for lamellipodia and filopodia formation respectively, and found upregulated in metastatic lesions, we further examined the activation status of Rac1 and Cdc42 in the absence or presence of EGF by G-LISA (Figure 2E). EGF stimulation significantly increased GTPase activity in Rac1 and Cdc42 in both cell lines. In the NCI-N87 cells JWA overexpression reduced GTPases activity (P<0.05). In HGC-27 JWA silencing significantly increased GTPases activity (P<0.05). The Cdc42 activity was nearly double that of Rac1 in control siRNA cells without EGF in HGC-27 cells, that could explain the more pronounced filopodia observed in HGC-27 cells (Figure 3H). Previous research has shown that p21-activated kinase 1 (PAK1) can be directly activated by the small GTPases Cdc42 and Rac1, or phosphorylated by Akt. By induction or silencing of JWA, PAK1 and p-PAK1 decreased and increased respectively as detected by western blotting (Figure 2F). Collectively, these data suggested that JWA regulates HER2/AKT signaling, resulting in F-actin remodeling and a diminished migratory ability of HER2-positive cells.

### The attenuation of cell motility by JWA is partly dependent on HER2

To confirm the effect of JWA on GC cell motility mediation by HER2, a full-length wild-type HER2 plasmid (HER2 WT) was used to re-express HER2 in JWA-overexpressing NCI-N87 cells. In response to concomitant expression of JWA and HER2, the JWA-induced suppression of migration was overridden by the rescued HER2 expression (Figure 3A, 3C). The enhanced motility after RNAi-mediated JWA silencing in HGC-27 cells was significantly attenuated by the HER2 inhibitor, CP714724, and by HER2 knockdown using shRNA (shHER2) (Figure 3B, 3D). F-actin staining revealed that the reduced lamellipodia (yellow arrows) induced by JWA expression began to stretch after HER2 re-expression in JWA-overexpressing NCI-87 cells (Figure 3G). In JWA-silenced HGC-27 cells, treatment with CP714724 or transfection of shHER2 markedly decreased the number of filopodia (white arrow) (Figure 3H). The JWA-associated reduction in the levels of p-AKT (Figure 3C) and Cdc42/Rac1 activity were reversed by rescuing HER2 expression in NCI-N87 cells (Figure 3E). Similarly, the activation of downstream AKT (Figure 3D) and Cdc42/Rac1 (Figure 3F) activity was abated by CP714724 or shHER2 in JWA-silenced HGC-27 cells, supporting the essential role of HER2 in the JWA-mediated suppression of cell motility.

### PEA3 is involved in JWA-mediated HER2 downregulation

**Figure 4:**
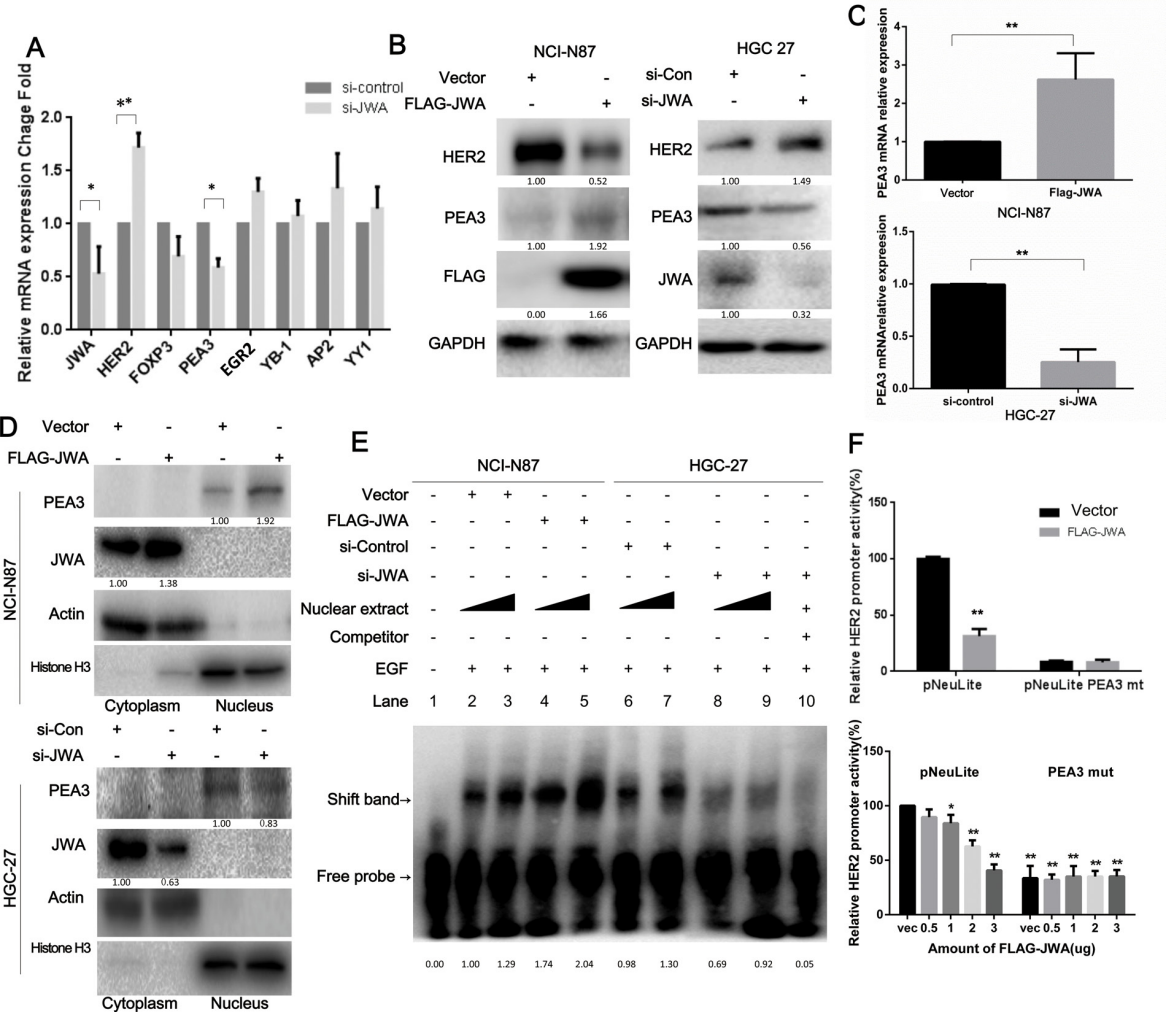
HER2 expression is modulated by JWA through PEA3 upregulation and activation **A.** The mRNA levels of HER2, JWA and a panel of putative transcription factors that regulate HER2 were determined by qPCR after HGC-27 cells were transfected with si-JWA and scramble control RNA. **B, C.** The mRNA and protein levels of HER2, JWA and PEA3 were identified by qPCR or western blot analyses in NCI-N87 cells transfected with FLAG-JWA or vector as well as in HGC-27 cells transfected with si-JWA or scramble control. * P<0.05; ** P<0.01; Student's t-test. **D.** PEA3 levels in nuclear and cytoplasmic extracts were confirmed by western blotting in JWA-overexpressing NCI-N87 cells and JWA-silenced HGC-27 cells. Actin and Histone H3 were used as cytoplasmic and nuclear loading controls, respectively. **E.** Four and six micrograms of nuclear protein were extracted from JWA-knockdown HGC-27 cells and JWA-overexpressing NCI-N87 cells to perform EMSA with a biotinylated oligonucleotide containing the PEA3-binding site and its competitive probe. **F.** NCI-N87 cells were transiently co-transfected with 2.5 μg (upper panel) or different amounts of FLAG-JWA (lower panel) and 2.5 μg of HER2 luciferase reporter promoter plasmids without (pNeuLite) or with the PEA3-binding site mutation (PEA3mut). The cells were lysed 36 h after transfection, and the luciferase activity was measured. The relative HER2 promoter activity was calculated relative to the activity of the wild-type promoter in vector cells (defined as 100%) after normalization to pRL-CMV. * P<0.05 and ** P<0.01 compared with vector pNeuLite activity.

Observing the parallel increase of the mRNA and protein levels of HER2 in JWA-silenced cells versus control cells (Figure 2C), suggested that HER2 could be regulated by JWA at the transcriptional level. Recent evidence has revealed that HER2 overexpression may depend on active gene transcription in the absence of gene amplification [23]. Several factors are involved in regulating HER2 transcription [24–30]. Therefore, the mRNA levels of well-established HER2 upstream transcription factors including AP-2, FOXP3, EGR2, YY1 and YB-1 were examined in JWA-silenced HGC-27 cells. The PEA3 mRNA expression decreased significantly after downregulating JWA (Figure 4A). Moreover, PEA3 mRNA and protein levels were increased in JWA-overexpressing NCI-N87 cells and decreased in JWA-silenced HGC-27 cells (Figure 4B, 4C). To determine if JWA can alter the subcellular localization of PEA3, nuclear and cytoplasmic lysates were analyzed by western blotting. Nuclear PEA3 expression was elevated in JWA-overexpressing NCI-N87 cells and reduced in JWA-silenced HGC-27 cells (Figure 4D). To further verify if JWA controls HER2 gene expression, dual-luciferase reporter assays were conducted. Co-transfection of a luciferase reporter driven by the wild-type (pNeuLite) HER2 promoter with FLAG-JWA into NCI-N87 cells resulted in suppressed HER2 promoter activity in a dose-response manner (Figure 4F, lower panel), but JWA failed to inhibit HER2 promoter activity when the PEA3-binding sequence was mutated (PEA3mut; 5′–AGGAAG–3′ to 5′–AGCTCG–3′; Figure 4F, upper panel) even if a larger amount of FLAG-JWA was added (Figure 4F, lower panel). To further determine if JWA influences PEA3-binding to a specific HER2 promoter region, electrophoretic mobility shift assays were performed. Ectopically expressed JWA increased PEA3 binding to the HER2 promoter, and loss of JWA had the opposite effect. These binding events were impeded by the addition of a 100-fold excess of a competitive probe (Figure 4E). In addition, the binding capacity significantly increased with the amount of nuclear extract only in JWA-overexpressing cells and not in JWA-deficient cells. These data further indicated that JWA regulates PEA3 expression and binding ability to the HER2 promoter, thus promoting HER2 transcriptional activity.

### The essential role of PEA3 in suppressing the motility by JWA

The role of PEA3 in suppressing the JWA-mediated motility of HER2-positive GC cells in the EGF-induced setting was studied by silencing PEA3 in JWA-transfected NCI-N87 cells, showing that cell migration significantly increased (Supplementary Figure S3A, left and middle panel). Additionally, the GTPase activity of Rac1 and Cdc42 increased 1.5-fold and 2-fold, respectively (Figure 4H), following PEA3 knockdown. In agreement with the phenotype, the loss of PEA3 prevented JWA-mediated HER2 downregulation (Supplementary Figure S3A, right panel). These data indicated that inhibition of cell migration by JWA is PEA3-dependent.

In two previous studies, we showed that JWA inhibited oncogenic transcript factor Sp1, which in turn inhibited the MMP-2-dependent angiogenic potential of endothelial cells in gastric cancer [17] and integrins αV and β3 in melanoma metastasis [9]. To further investigate if PEA3 inhibition by JWA observed in the present study is linked to JWA-regulated Sp1-related-pathways, knockdown experiments of Sp1 were conducted in HGC27 cell lines. Silencing Sp1 showed significantly increased cell migration as well as small GTPase activity (Supplementary Figure S3D), and significant influence on Sp1 downstream MMP2 level, but non-significant impact on PEA3 expression in the JWA-deficient HGC-27 cells (Supplementary Figure S3E). Similarly, downregulating PEA3 increased the migratory potential and decreased small GTPase activity (Supplementary Figure S3A, S3B) but had no influence on Sp1 and its downstream MMP2 in the JWA-over-expressing NCI-N87 cells (Supplementary Figure S3C). The results show that, the PEA3/HER2 may be a Sp1-independent signalling pathway in JWA-mediated metastasis suppression.

### ERK activation is required for the regulation of PEA3/HER2 by JWA

**Figure 5:**
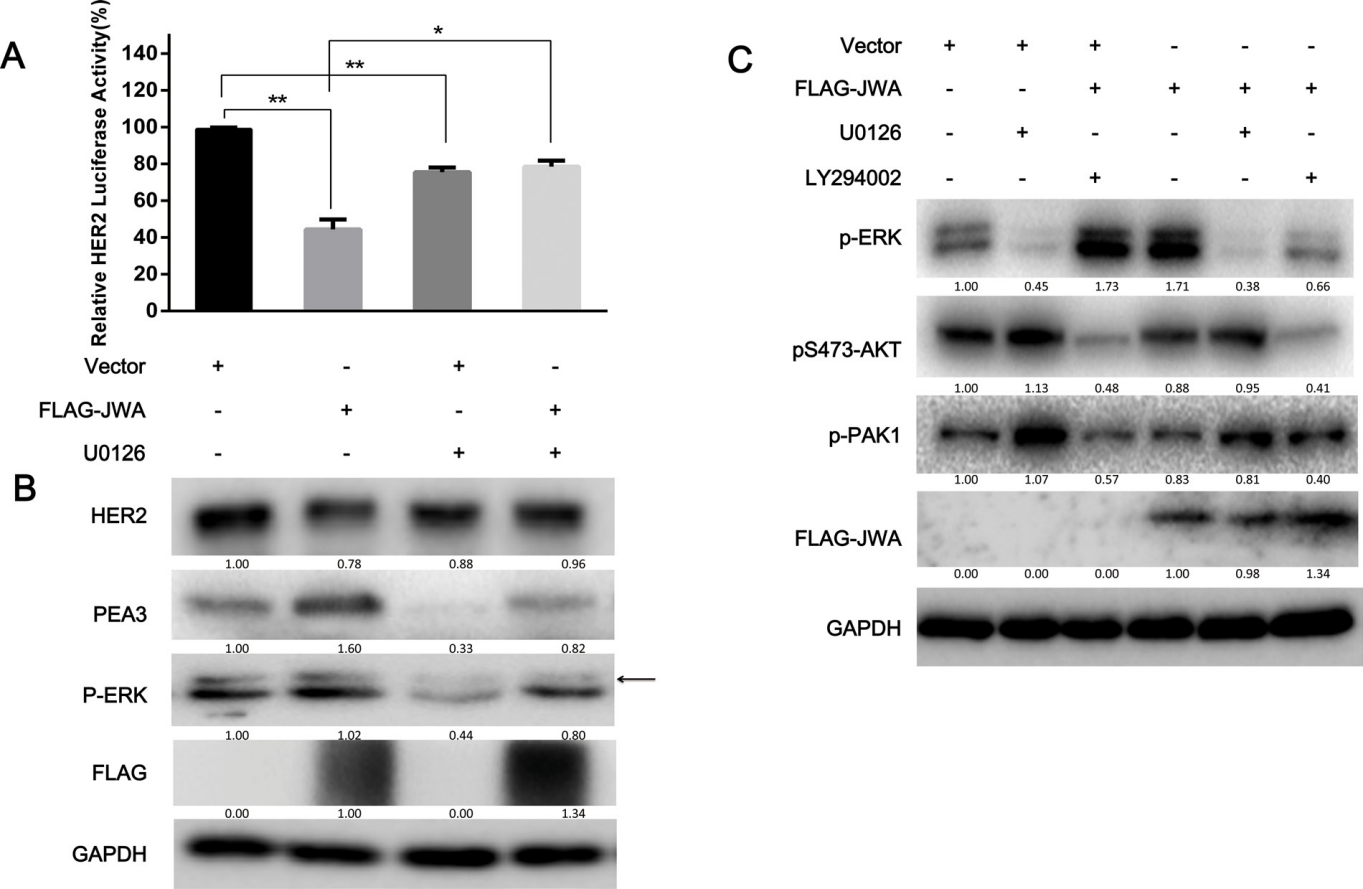
ERK phosphorylation is required for the JWA/PEA3 signaling axis **A, B.** NCI-N87 cells were transfected with FLAG-JWA or vector for 36 h and then treated with U0126 (25 μg/ml) or DMSO for 6 h and EGF (100 ng/ml) for 20 min. HER2 luciferase reporter promoter plasmids (pNeuLite) were co-transfected with vector or FLAG-JWA into NCI-N87 cells. The luciferase activity was normalized to pRL-CMV. The HER2 promoter luciferase activity in vector-treated cells was used as a control to calculate the relative HER2 luciferase activity (A). The PEA3, p-ERK, JWA, and HER2 protein levels were examined by western blot analysis (B). **C.** The MEK (U0126; 25 μM) and/or PI3K (LY294002; 50 μM) inhibitors or control DMSO were added to the cells for 6 h, and the cells were then transfected with FLAG-JWA or vector for 36 h. The cells were then harvested to detect HER2, p-AKT, p-ERK, p-PAK1, FAK, COX2, or FLAG by immunoblotting. The data in the bar graphs represent the mean and SD of three independent experiments. * P<0.05 and ** P<0.01; Student's t-test. Arrows indicate the interested band.

Research has shown PEA3 associated to the MEK/ERK signaling pathway and growth factor/ERK signaling in melanoma and gastrointestinal stromal tumors [31, 32]. Therefore, we hypothesized that the PEA3 regulation by JWA occurred through activating MAPK cascades. To test this hypothesis, JWA-overexpressing NCI-N87 cells were treated with the MEK inhibitor U0126, or DMSO for 4 h. The U0126 significantly decreased PEA3 expression (Figure 5B) and transcriptional activity (Figure 5A), supporting the vital role of ERK signaling in maintaining PEA3 expression. Moreover, the repressive activity of JWA on HER2 levels was significantly reversed by U0126, suggesting that JWA modulate the PEA3/HER2 axis via ERK activation.

It has been shown that the PI3K/AKT and MEK1/2/ERK1/2 pathways control cell survival and migration via crosstalk [33]. Therefore, we investigated whether JWA also could lead to AKT activation via p-ERK outside of the p-MEK/PEA3/HER2/p-AKT pathway. Inhibition of MEK with U0126 increased AKT phosphorylation, which was not significantly modulated by JWA, whereas PI3K inhibition with LY294002 significantly repressed p-ERK levels in JWA-overexpressing NCI-N87 cells (Figure 5C). These data suggested that JWA can modulate cell motility in a PEA3/HER2-dependent or PEA3/HER2-independent manner. This result also provides evidence for a partial role of HER2 in the suppression of cell motility by JWA.

The MEK/ERK is one of the main pathways downstream of HER2 [34]. The impact of HER2 on MEK/ERK pathways was examined by p-ERK level detection with western blot after treatment with HER2 inhibitor CP724714 in JWA-silenced HGC27 cells (Figure 3D) and after rescued HER2 expression in JWA-overexpressing NCI-N87 cells (Figure 3C). The data showed HER2 expression increases MEK/ERK activation, but is dependent of HER2 repression by JWA on MEK/ERK activation. Therefore, HER2/ERK forms a negative feedback loop in the network to regulate the pathway activation.

In a previous study, we showed that JWA could function as a kinase downstream of c-raf and upstream of MEK via the Ser138 motif in As_2_O_3_-inhibited and PMA-induced cell motility [15]. In this study, JWA could activate MEK/ERK pathway but did not influence Raf activation (Supplementary Figure S2A, S2B), which was consistent with our previous result in melanoma [9, 15]. Immunoprecipitation was performed in FLAG-JWA-transfected cells to explore whether JWA could interact with MEK/ERK directly. Interestingly, Flag-JWA could interact with B-raf, p-MEK, MEK but not with c-raf, ERK, p-ERK (Supplementary Figure S4A). In addition, JWA also increases the interaction of B-raf with p-MEK (Supplementary Figure S4B). These data indicated that JWA seems to be essential for MEK–MAPK signaling activation in a direct pattern. Collectively, JWA could enhance PEA3 activity and consequently inhibit HER2 expression via interacting and activating MEK directly. In turn, declined HER2 level could decrease MEK/ERK activation to maintain the balance of the HER2/MEK/ERK loop.

### JWA has little impact on HER2 expression and activation in HER2-negative BGC823 cells

When we examined the association of JWA with HER2 in HER2-negative BGC-823 cells, with moderate JWA expression, JWA had little effect on HER2 expression and activation (Supplementary Figure S5B) even though it still affected migration (Supplementary Figure S5A). However, when HER2 and JWA were co-expressed in BGC-823 cells, JWA exerted a pronounced inhibitory effect on EGF-induced migration (Supplementary Figure S6A) and HER2 expression, and JWA enhanced PEA3 expression similar to the enhancement induced by EGF and HER2 overexpression (Supplementary Figure S6B). Moreover, the extent of HER2 downregulation and PEA3 upregulation increased with increasing exogenous HER2 expression (Supplementary Figure S6C, S6D), suggesting that the modulation of HER2 by JWA may be dependent on cellular HER2 and PEA3 levels.

### JWA is reduced in GC tissues, and JWA loss is negatively related to high HER2 expression

**Figure 6:**
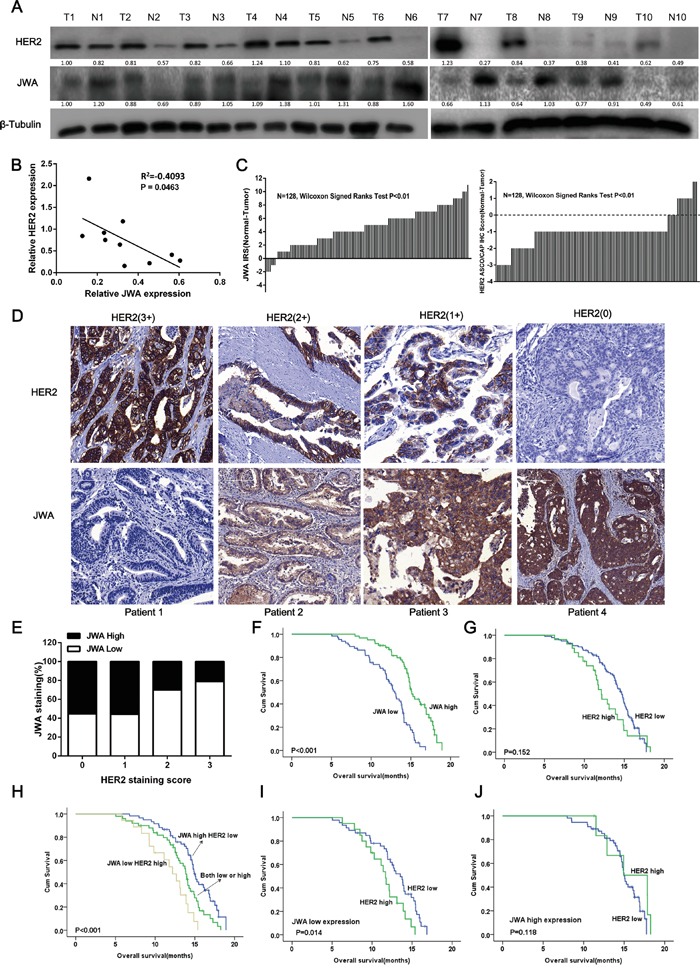
JWA is negatively associated with HER2 expression in gastric cancer (GC) tissue, and low JWA predicts a poor prognosis and stratifies a high-risk subgroup of HER-2-positive advanced gastric cancer (AGC) **A.** Gastric cancer tissues and adjacent noncancerous normal gastric tissues from 10 different patients were lysed. The lysates were probed for JWA and HER2. β-Tubulin was used as a loading control. **B.** Correlation analysis of the relationship between relative JWA protein expression and relative HER2 expression in 10 GC tissues. **C.** The distribution of the JWA and HER2 staining scores. JWA and HER2 expression was examined by IHC in 128 paired human GC and adjacent normal tissues (Wilcoxon's signed-rank test, P<0.01). The data are presented as the difference between the noncancerous score and the paired cancer score (including the JWA IRS and the HER2 ASCO/CAP IHC score). **D.** Representative images of JWA and HER2 immunohistochemical staining in human gastric cancer lesions with different HER2 scores. Scale bars, 100 μm. **E.** The negative rate of JWA across all HER2 scores (n=128, Chi square test, P<0.01). **F-J.** Kaplan–Meier analysis of the overall survival (OS) of AGC patients based on JWA (F, high JWA expression, n= 62; low JWA expression, n= 66), HER2 (G, high HER2 expression, n= 27; low HER2 expression, n= 101) or combined JWA/HER2 (H, high JWA/low HER2, n= 52; both high or both low, n= 58; low JWA/high HER2, n= 18). P values were calculated using the log-rank test. **I, J.** Kaplan-Meier plot illustrating the OS based on HER2 expression of AGC patients with high JWA expression (I) or low JWA expression (J).

To investigate the expression of JWA and HER2 in GC tissue, immunoblotting was performed on human fresh tumor and adjacent normal tissue samples from patients with gastric adenocarcinoma (n=10), and immunohistochemical (IHC) staining was performed on a tissue microarray (TMA) of local advanced or metastatic GC patients, including GEJ, (n=128) to detect HER2 and JWA. According to the ROC analysis, the optimal cutoff for the JWA IRS was 4. Thus, IRS 0-3 and 4-12 indicated low and high JWA expression, respectively. Compared with normal gastric tissue, 9/10 (90%) and 115/128 (89.8%) of the GC cases exhibited increased HER2 expression, and 8/10 (71.6%) and 121/128 (94.5%) of the GC cases had lower JWA expression in the fresh samples (Figure 6A) and the TMA (Figure 6C), respectively. Negative correlations were also observed between HER2 and JWA in both fresh tissue (P<0.05, Figure 6B) and the TMA (P<0.01, Table 1).

**Table 1:** Relationship between expression levels of JWA, HER2 and clinicopathologic characteristics of the advanced gastric cancer cohort (n=128)

Variables	JWA expression				P value	HER2 expression				P value
	Low, n (%)		High, n(%)			Low, n(%)		High, n(%)		
All (N)	66		62			101		27		
Age (years)										
≤57	32	(48.5)	38	(61.3)	0.159	59	(58.4)	11	(40.7)	0.129
>57	34	(51.5)	24	(38.7)		42	(41.6)	16	(59.3)	
Gender										
Males	41	(62.1)	42	(67.7)	0.58	67	(66.3)	16	(59.3)	0.504
Females	25	(37.9)	20	(32.3)		34	(33.7)	11	(40.7)	
Disease status										
Metastatic diseases	11	(16.7)	12	(19.4)	0.819	20	(19.8)	3	(11.1)	0.403
Locally advanced	55	(83.3)	50	(80.6)		81	(80.2)	24	(88.9)	
Depth of invasion										
T1/T2	8	(12.1)	10	(16.1)	0.614	15	(14.9)	21	(77.8)	0.63
T3/T4	58	(87.9)	52	(83.9)		86	(85.1)	6	(22.2)	
Tumor diameter(cm)										
≤5	21	(31.8)	17	(27.4)	0.699	30	(29.7)	3	(11.1)	0.763
>5	45	(68.2)	45	(72.6)		71	(70.3)	24	(88.9)	
Primary Location										
GEJ	31	(47.0)	17	(27.4)	0.029	34	(33.7)	8	(29.6)	0.994
stomach	35	(53.0)	45	(72.6)		67	(66.3)	19	(70.4)	
Histologic type										
Intestinal	32	(48.5)	43	(69.4)	0.02	52	(51.5)	14	(51.9)	0.116
Diffuse/mixed	34	(51.5)	19	(30.6)		49	(48.5)	13	(48.1)	
Grading										
G1/G2	35	(53.0)	28	(45.2)	0.384	48	(47.5)	23	(85.2)	0.002
G3	31	(47.0)	34	(54.8)		53	(52.5)	4	(14.8)	
Lymph node metastasis										
N0/N1	13	(19.7)	36	(58.1)	<0.001	48	(47.5)	15	(55.6)	0.519
N2/N3	53	(80.3)	26	(41.9)		53	(52.5)	12	(44.4)	
Peritoneum metastasis										
Negative	48	(72.7)	55	(88.7)	0.027	82	(81.2)	1	(3.7)	<0.001
Positive	18	(27.3)	7	(11.3)		19	(18.8)	26	(96.3)	
Liver metastasis										
Negative	14	(21.2)	41	(66.1)	<0.001	57	(56.4)	21	(77.8)	0.785
Positive	52	(78.8)	21	(33.9)		44	(43.6)	6	(22.2)	
Number of metastasis organs										
=1	48	(72.7)	52	(83.9)	0.14	82	(81.2)	4	(14.8)	<0.001
>1	18	(27.3)	10	(16.1)		19	(18.8)	23	(85.2)	
ECOG										
0-1	32	(48.5)	40	(64.5)	0.077	60	(59.4)	18	(66.7)	0.12
2	34	(51.5)	22	(35.5)		41	(40.6)	9	(33.3)	
Previous gastrectomy										
No	43	(65.2)	50	(80.6)	0.073	72	(71.3)	12	(44.4)	0.193
Yes	23	(34.8)	12	(19.4)		29	(28.7)	15	(55.6)	
Chemotherapy agents										
Doublet/Single	49	(74.2)	48	(77.4)	0.686	75	(74.3)	22	(81.5)	0.614
Triplet	17	(25.8)	14	(22.6)		26	(25.7)	5	(18.5)	
HER2 expression										
Low	46	(69.7)	55	(83.3)	0.01	---		---		---
High	20	(30.3)	7	(10.6)		---		---		---

GEJ, Gastroesophageal junction; ECOG, Eastern Cooperative Oncology Group.

Representative IHC images of tissue samples with different HER2 staining scores that were stained with the JWA and HER2 antibodies are presented in Figure 6D. In cases with high intensity membrane HER2 staining (3+ or 2+), JWA exhibited little cytoplasmic expression (IRS 0 and 4, respectively), whereas for patients with low HER2 expression (1+ or 0+), the JWA staining was much stronger (IRS 8 and 12, respectively). Furthermore, the rate of JWA negativity increased with increasing HER2 IHC scores, but the trend was not significant in samples with HER2 IHC scores of 1+ or 0+ (Figure 6E).

### JWA/HER2 expression and clinicopathological variables in GC

The relationship between JWA or HER2 expression and clinicopathologic features were further analyzed in the AGC TMA (Table 1). JWA and HER2 expression shared similar significant associations with histological type (P=0.020 and 0.002, respectively), lymphatic metastasis (both P<0.001) and liver metastasis (both P<0.001). In addition, low JWA expression was more frequent in tumors with peritoneal metastasis (P=0.027) and stomach-derived metastasis (P=0.029). However, no differences were observed with respect to other clinicopathological characteristics.

### Combined low JWA and high HER2 expression predicts a poor outcome for advanced gastric cancer patients, and low JWA stratifies a subgroup of HER2-positive patients with poor outcome

To clarify the prognostic value of JWA and HER2 in AGC independently and in combination, we performed further analyses of the TMA cohort with a median follow-up of 20 months (range 5-28 months) and 98 deaths (76.6%). Low JWA expression in the tumors significantly correlated with shorter OS (P<0.001). Both univariate and multivariate analyses (Table 2) indicated that low JWA expression was an independent negative prognostic factor for AGC (adjusted HR, 0.564; P=0.024, Figure 6F). HER2-positive patients had a similar OS as patients with HER2-negative disease (P=0.152, Figure 6G), and HER2 overexpression was not an independent predictor of survival (HR, 1.448; P=0.119; Table 2). Intriguingly, when the patients were divided into 3 groups based on the staining score for JWA and HER2 (both high or both low, n=50; high JWA and low HER2 low, n=60; low JWA and high HER2, n=18), patients with low JWA and high HER2 had a worse outcome than the other two groups (P <0.001, Figure 6H). After adjusting for other risk factors, the combination of JWA and HER2 was still a powerful prognostic marker for AGC (both low or both high vs high JWA/low HER2: HR=0.442, P=0.024; both low or both high vs low JWA/high HER2: HR=0.323, P=0.006). When the patients were stratified by JWA expression, those with higher HER2 expression exhibited a significantly shorter OS than those with lower HER2 expression in the low JWA subgroup (P=0.0014, Figure 6I). However, no significant differences in OS were observed between the HER2-positive and HER2-negative groups when JWA expression was high (P=0.118, Figure 6J).

**Table 2:** Cox regression analyses of JWA, HER2 and clinicopathologic variables for overall survival in the advanced gastric cancer (n=128)

Variables	P	HR	95% CI	
			Lower	Upper
**Univariate analysis**				
Age (years)(>57 vs ≤57)	0.539	1.134	0.760	1.692
Gender(Males vs Females)	0.194	1.316	0.870	1.990
Disease status(Metastatic vs Local advanced)	0.417	1.258	0.723	2.188
Depth of invasion(T3/T4 vs T1/T2)	0.656	1.134	0.652	1.973
Tumor diameter(cm)(>5 vs ≤5)	0.861	0.961	0.614	1.503
Primary Location (GEJ vs Mid to distal stomach)	0.704	0.924	0.613	1.392
Histologic type(Diffuse/mixed vs Intestinal)	<0.001	2.793	1.818	4.291
Grading(G3 vs G1/G2)	0.134	1.359	0.909	2.030
Lymph node metastasis(N2/N3 vs N0/N1)	0.001	2.107	1.355	3.278
Peritoneum metastasis(positive vs negative)	<0.001	2.894	1.745	4.800
Liver metastasis(positive vs negative)	<0.001	2.104	1.392	3.178
Number of metastasis organs(>1 vs 1)	0.097	1.466	0.933	2.303
Performance status (ECOG)(2 vs 0-1)	<0.001	2.606	1.718	3.953
Previous gastrectomy(Yes or No)	0.404	1.200	0.782	1.841
Chemotherapy agents(Triplet vs Doublet/Single)	0.092	0.654	0.399	1.072
HER2 expression(high vs low)	0.119	1.448	0.909	2.306
JWA expression(low vs high)	<0.001	2.463	1.597	3.788
JWA/HER2 expression				
(JWA high HER2 low vs. both low or high)	<0.001	0.281	0.153	0.516
(JWA high HER2 low vs JWA low HER2 high)	0.009	0.457	0.253	0.824
**Multivariate analysis**				
JWA				
Performance status (ECOG)(2 vs 0-1)	<0.001	2.257	1.431	3.558
Histologic type(Diffuse/mixed vs Intestinal)	0.002	2.033	1.291	3.202
Lymph node metastasis(N2/N3 vs N0/N1)	0.030	1.722	1.053	2.816
Peritoneum metastasis(positive vs negative)	0.001	2.527	1.463	4.365
Liver metastasis(positive vs negative)	0.153	1.413	0.880	2.268
JWA expression(low vs high)	0.024	1.773	1.079	2.915
JWA/HER2				
Performance status (ECOG)(2 vs 0-1)	0.003	2.081	1.289	3.360
Histologic type(Diffuse/mixed vs Intestinal)	<0.001	2.774	1.667	4.614
Lymph node metastasis(N2/N3 vs N0/N1)	0.051	1.670	0.998	2.794
Peritoneum metastasis(positive vs negative)	0.002	2.425	1.386	4.244
Liver metastasis(positive vs negative)	0.405	1.241	0.746	2.065
JWA/HER2 expression				
(JWA high HER2 low vs. both low or high)	0.024	2.262	1.112	4.608
(JWA low HER2 high vs JWA high HER2 low)	0.006	3.096	1.395	6.897

GEJ, Gastroesophageal junction; ECOG, Eastern Cooperative Oncology Group; HR, hazard ratio; CI, confidence interval.

## DISCUSSION

In this study we demonstrated a novel mechanism of direct negative regulation of HER2 expression by JWA affecting the cytoskeletal rearrangement and motility of HER2 positive cells, and that combined low JWA and high HER2 expression are negative prognostic of the HER2 positive subgroup of gastric cancer.

Overexpression of HER2 mediates an aggressive tumor phenotype and a high metastatic risk in several cancers [21, 34]. Tumor stroma-derived epidermal growth factor (EGF), one of its most crucial ligands, has been implicated in this metastatic potential, predominantly in terms of cell migration through downstream signaling pathways, such as the MAPK/ERK and PI3K/AKT pathways [22], and EGF-induced chemotaxis of invasive cells to blood vessels is an essential step of metastasis.

JWA is a microtubule-associated protein (MAP), that co-localize with tubulin and has been demonstrated to enhance As_2_O_3_-induced apoptosis in HeLa and MCF-7 cancer cells by promoting tubulin polymerization [35]. Moreover, JWA is a repressor of multiple stages of metastasis, such as cellular adhesion, invasion and angiogenesis, in different types of cancer [9, 12–15, 17]. As a MAP, JWA also promotes an altered F-actin distribution in response to As_2_O_3_ and PMA [15]. However, the mechanism by which JWA regulates cell motility has not been clearly elucidated, especially in HER2-positive disease.

Most of the suppressive effects of JWA on metastasis have been reported based on the transcriptional and post-transcriptional regulation of Sp1, e.g., downregulating MMP2 protease expression to compromise angiogenesis in GC or decreasing Integrin αVβ3 expression to impair adhesion and angiogenesis in melanoma. Here, PEA3 was found to be responsible for JWA-mediated HER2 downregulation in GC. The PEA3 has been identified as a negative transcription factor that binds to the HER2 promoter to inhibit the growth and progression of HER2-overexpressing ovarian and breast cancer cells [36]. Another supporting study has reported that inhibiting fatty acid synthetase (FAS) downregulates HER2 expression by upregulating PEA3 in breast cancer cells [37]. However, contradictory results suggest that PEA3 functions as an oncoprotein to activate the HER2 promoter with p-300 and is required for mammary oncogenesis [38, 39]. In our study, PEA3 played an inhibitory role in cell migration by inhibiting HER2 promoter activity in HER2-positive GC cell lines but not in HER2-negative BGC-823 cells. This inhibition may have been due to the interaction of PEA3 with other PEA3 subfamily members, namely ER81/ETV1 and ERM/ETV5, which share a highly conserved ETS domain that determines DNA-binding specificity [40] and may positively regulate the promoters of HER2 and many other invasion-related genes. In agreement with previous studies, we found that the activity of the PEA3 site-mutated HER2 promoter was significantly lower than that of the wild-type HER2 promoter in the reporter gene assay, implying that the PEA3-binding motif in the HER2 promoter is a positive regulatory element for HER2 gene transcription. Therefore, PEA3 may have relatively lower transcription efficacy but higher binding affinity to compete for the motif with other transactivation factors in the Ets families, thus inhibiting HER2 expression in HER2-positive cells rather in HER2-negative cells.

Notably, it has been reported that PEA3 serves as a negative regulator of HER2 only in HER2-positive cells. This is consistent with our observation that JWA can modulate HER2 expression only in HER2-overexpressing BGC-823 cells with an elevated PEA3 expression, and the extent of inhibition increases with the level of HER2 overexpression. Furthermore, the evidence from the AGC TMA supported the results that the percentage of negative JWA tissues was higher among those with strong HER2 staining (IHC score 3+) than among those with an IHC score of 2+, 1+ or 0+. However, no significant difference was observed between HER2 1+ and 0+ patients.

In our previous study, As_2_O_3_ and PMA modulated cell migration mainly via activating the downstream MAPK/ERK pathways [22]. We found that JWA promotes MEK/ERK activation directly but hinders AKT activation, which was consistent with the decreased migration. Theoretically, MAPK/ERK, as the HER2 downstream signaling pathway, should be abrogated if HER2 expression is reduced by JWA. However, EGF mainly activates AKT signaling to promote cell motility as its effect on ERK1/2 phosphorylation was less pronounced (data not shown). This result corresponded with the reports that invasive cells constitute a subpopulation that is neither proliferative nor apoptotic but is instead highly motile [7]. ERK1/2 is primarily responsible for cell proliferation, while PI3K/AKT predominantly influences cell motility and survival. Thus, for motile cancer cells, PI3K/AKT signaling is activated more easily in response to EGF. Therefore it is reasonable that JWA greatly enhanced MAPK/ERK activation, which may have offset HER2-induced ERK activation.

It is well established that MAPK/MAPKAPK phosphorylation upregulates PEA3 expression and enhances PEA3 transcriptional activity. In our previous study, JWA protein harbors 2 PKC phosphorylation-binding sites, namely Ser18 (SDR) and Ser138 (SLR). Ser138 mutation blocks the activation of MEK and ERK, while Ser18 mutation does not block this activation. In addition, these mutations may have little impact on the activation of c-raf, suggesting that JWA may be a kinase downstream of c-raf and upstream of MEK via the Ser138 (SLR) motif during As_2_O_3_-inhibited and PMA-induced cell motility [15]. This partially explained the molecular mechanism underlying PEA3 activation by JWA. Interestingly, we found JWA could interact with molecules in MEK–MAPK signaling to mediate its activation. This finding and mechanism is being further investigated by our team and results to be published later.

For breast cancer (BC) patients, HER2 overexpression is associated with significantly worse survival compared with HER2 negativity. Nevertheless, in GC, the prognostic significance of HER2 remains controversial [3, 41–44]. Recently, it has been demonstrated that JWA has prognostic value independent of or in combination with XRCC1 [10], P53 [45], FAK [14] or MDM2 in resected GC patients. The significance of JWA in HER2 regulation in HER2-positive GC cells prompted us to evaluate its potential clinical application in subpopulations who have never received trastuzumab treatment because trastuzumab treatment in combination with chemotherapy has shown significant survival benefits for patients with HER2 positive AGC. An inverse correlation between JWA and HER2 expression was detected in the cohort. Consistent with some studies [42, 44], our data showed that HER2 was not a prognostic factor for AGC patients. Intriguingly, concomitant expression of low JWA and high HER2 was closely related to the overall survival of patients with AGC. After stratifying by JWA expression, HER2 positivity was significantly associated with poor prognosis only in the low JWA expression group. Therefore, more intensive treatment and more frequent follow-up appointments should be considered by oncologists for AGC patients with low JWA and high HER2 expression, whereas high-JWA patients and HER2-negative AGC patients could be treated with less cytotoxic agents.

**Figure 7:**
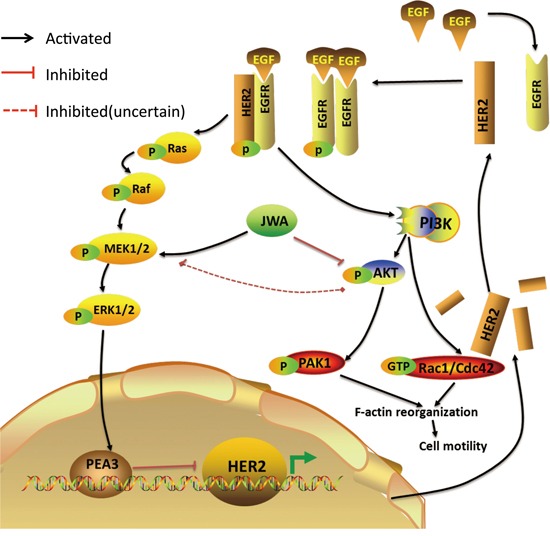
Proposed model of the JWA-mediated regulation of HER2 JWA enhances PEA3 expression and its negative transcriptional activity on the HER2 promoter by activating MEK/ERK, resulting in HER2 downregulation. A decreased HER2 level, which attenuates HER2 downstream PI3K/AKT signaling, Rac1/Cdc42 activation, PAK1 activation and F-actin-based pseudopod stretching, represses cell motility in HER2-positive gastric cancer cells.

Taken together, our results demonstrate that JWA is a novel negative regulator of HER2 expression as well as a negative regulation of EGF-induced cell migration and cytoskeletal changes in HER2-positive GC cells through a previously unrecognized mechanism involving the PEA3 transcription factor. JWA enhanced PEA3 expression and its negative transcriptional activity on the HER2 promoter by activating MEK/ERK signaling, resulting in HER2 downregulation, downstream PI3K/AKT attenuation and consequent inhibition of Rac1/Cdc42 activation and pseudopod stretching (Figure 7). JWA may also be a promising prognostic marker for AGC to help stratify HER2-positive subgroups to better identify unfavorable outcomes. These results indicate a potential clinically important role of JWA/HER2 as a prognostic biomarker, but still needs to be verified in prospective studies.

## MATERIALS AND METHODS

### Patients and specimens

A total of 128 advanced gastric cancer (AGC) and paired non-cancerous patient tissues were provided by the National Biochip Engineering Research Center (Shanghai, China) from 1 April 2008 to 30 Sep 2012 to develop tissue microarrays (TMAs). The inclusion criteria were advanced or metastatic gastric or gastroesophageal junction (GEJ) carcinoma with available tumor tissue. The exclusion criteria were resectable gastric cancer or active benign gastric disease. Furthermore, ten pairs of histology-confirmed gastric cancer and adjacent normal fresh gastric mucosa tissues were obtained from Nantong Cancer Hospital. Western blot was then employed for determining HER2 and JWA expression in tissue lysates as previously described [16]. Written informed consent was obtained from the subjects prior to obtaining tissue. This study was approved by the Research Ethics Committee of the corresponding hospitals.

Detailed clinicopathologic information was obtained from patient records. The 7th TNM stage and Lauren subtype were independently verified by two pathologists. Overall survival (OS) was the primary endpoint, which was calculated from the date of pathological diagnosis of gastric cancer to the date of death or the last follow-up. The date of death for each case was verified using patient records and the public security department. Follow-up was conducted by telephone calls until June 2014.

The TMA was constructed, and immunohistochemistry (IHC) was performed as described previously with the antibodies listed in Supplementary Table S2. JWA and HER2 staining was assessed by two independent pathologists blinded to patient history. HER2 was evaluated based on the current ASCO/CAP IHC assessment criteria [16]. Tumors with a HER2 score of 3+ or 2+ were considered HER2-positive, and those with a score of 0 or 1+ were considered HER2-negative. The immunoreactive score (IRS) was calculated for the JWA staining analysis by multiplying the staining intensity by the percentage of HER2-positive tumor cells as described previously [12]. The cutoff of the JWA IRS was determined according to receiver-operator characteristic (ROC) analysis [10], which has the best discriminative ability for predicting the 1-year survival rate.

### Cell lines and culture conditions

The AGS and NCI-N87 gastric cell lines (American Type Culture Collection, USA) as well as the HGC-27, SGC-7901, BGC-823, MGC-803, MKN-45 and GES-1 gastric cell lines (Shanghai Institute of Biochemistry and Cell Biology, Chinese Academy of Sciences) were cultured in RPMI 1640 medium supplemented with 10% fetal calf serum (FBS; Gibco, USA), 100 U/ml penicillin and 100 μg/ml streptomycin and were maintained at 37°C and 5% CO_2_ in a humidified atmosphere.

### Plasmids, small interfering RNA and reagents

The siRNA targeting JWA (5′-CGAGCTATTTCC TTATCTC-3′) and a scrambled control siRNA (RiboBio, Guangzhou, China) were transfected into cells using Lipofectamine 2000 (Invitrogen, Grand Island, USA). A HER2 short hairpin RNA (shRNA) (GenePharma, China) was constructed by subcloning the siRNA expression cassettes into the pGPU6/GFP/Neo vector. The origins of the Vector and FLAG-JWA plasmids have been previously described [17]. The HER2 wild-type (HER2 WT) construct was obtained from Mien-Chie Hung (Addgene, INSERT CITY, USA). The plasmids were transiently transfected into NCI-N87 cells using the Lipofectamine 3000 transfection reagent (Invitrogen, USA) according to the manufacturer's protocol. Her2 inhibitor CP724714, MEK-inhibitor U0126 and PI3K inhibitor LY294002 were purchased from Selleck Chemicals (Houston, USA). Epidermal Growth Factor (EGF) was purchased from PeproTech (Rocky Hill, USA).

### Cell migration assay

Cells were seeded on a fibronectin-coated polycarbonate membrane insert in a permeable transwell in 200 μl of RPMI 1640 medium with 1% FBS (Costar, MA, USA), and 600 μl of RPMI 1640 with 10% FBS and 100 ng/ml EGF was added to the lower compartment. After the cells were incubated for 10 or 24 h, the insert was washed with phosphate buffered saline (PBS), and the cells adhering to the lower surface were fixed with methanol for 15 min, stained with crystal violet for 10 min, and rinsed with PBS. The cells on the top surface were removed using a cotton swab. The cells were counted in five microscope fields (×200). All of the assays were independently repeated at least three times.

### G-LISA activation assay

To measure the Rho GTPase activation of Cdc42 and Rac1, cells were serum-starved overnight and then treated with 100 ng/ml EGF for 10 min. The cell lysates were analyzed using Cdc42 and Rac1 G-LISA Activation Assay Kits (Colorimetric Based) according to the manufacturer's instructions (Cytoskeleton, INSERT CITY, USA). The absorbance values were obtained at 490 nm. The measurements were performed in triplicate.

### Western blotting

The recently collected fresh GC tissues were ground into extracts as previously described. The whole cell or tissue extracts were prepared with RIPA lysis buffer [17] and protease inhibitor cocktail (Roche, Manheim, Germany). Nuclear extracts were obtained using the NE-PER Nuclear and Cytoplasmic Extraction Reagents (Pierce Biotechnology, Rockford, USA) according to the manufacturer's protocol. Western blotting was performed as previously described [17]. All of the antibodies used for Western blotting are presented in Supplementary Table S2. Each blot was repeated in triplicate. All the Western blots with untreated controls were listed in Supplementary Figure S1.

### Immunoprecipitation

HGC-27 cells were transfected for 24 h with Flag-JWA using Lipofectamine 2000 as described previously [18]. Cells were lysed in 20 mM Tris (pH 8.0), 137 mM NaCl, 10% glycerol, and 0.2% Triton X-100. Anti-B-Raf (Signal way Antibody, US) monoclonal antibodies or anti-Flag monoclonal antibodies were incubated with protein G- or protein A-Sepharose beads (Santa Cruz, Santa Cruz, USA) for 2 h at 4°C, washed, and incubated for 3 h at 4°C with equal amounts of precleared cell lysate. Non-immune mouse or rabbit serum (Sigma, St. Louis, USA) were used as controls. Samples were resolved by SDS/PAGE and Western blotting and probed with anti-b-raf, p-b-raf, c-raf, p-c-raf, p-MEK, MEK, p-ERK, ERK antibodies.

### Dual luciferase reporter assay

Cells were plated in 12-well plates and transfected with 1.0 μg of the pGL2 basic luciferase vector (Promega, Madison, USA), pNeuLite or pNeuLite PEA3 mt. The pNeuLite plasmids contain the core promoter of the HER2 gene, and the PEA3 mt plasmids have a mutated PEA3-binding motif in the HER2 promoter (kindly provided by Prof. Mien-Chie Hung; Addgene, MA, USA). The Renilla plasmid (Promega, Madison, USA) was co-transfected into the cells as an internal control. The cells were lysed in passive lysis buffer after a 24-h incubation. Luciferase activity was measured using the dual-luciferase reporter assay system. The measurements were performed in triplicate.

### Electrophoretic mobility shift assay

Electrophoretic mobility shift assays were performed using a Biotin Gel Shift Kit (Pierce Biotechnology, MA, USA) with nuclear extracts and a double-stranded oligonucleotide (5′-GGAGCTCGAGGGCTGCTTGAGGAAGTATAAGAATG-3′; Invitrogen, China) that was labeled with biotin at the 5′ end, and an oligonucleotide with the same sequence but lacking biotin was used as a competitive probe. In brief, 4-6 μg of nuclear extract was mixed with 200 fmol 5′ biotin-labeled, double-stranded probe bearing the PEA3 consensus binding sequence in 20 μl of the binding system. Competition reaction mixtures contained a 100-fold excess of non-labeled, double-stranded oligo-DNAs. The detection procedure was completed as previously described [17].

### RNA extraction and quantitative real-time PCR (qRT-PCR)

Total RNA was extracted from cells using TRIzol (Invitrogen, USA) and reverse-transcribed using a PrimeScript RT Reagent Kit (TaKaRa Bio, Japan) according to the manufacturer's instructions. Quantitative SYBR Green PCR assays were performed in an ABI Prism 7900 Sequence detection system (Applied Biosystems, Canada) using the SYBR Green Kit supplied by TaKaRa under previously described reaction conditions [19]. The primer sequences are presented in Supplementary Table S1. The target gene expression levels were normalized to those of GAPDH. The specificity of the amplified products was verified by melt curve analysis and agarose gel electrophoresis. The measurements were repeated in triplicate.

### Immunofluorescence and F-actin staining

Cells were seeded in 35-mm glass-bottom dishes and then fixed with a 3.7% paraformaldehyde solution, permeabilized with Triton X-100 and blocked in a 10% goat serum blocking solution in PBST for 1 h at room temperature before staining overnight with the antibodies listed in Supplementary Table S2. All of the antibodies were diluted in goat serum blocking solution/PBST. After the dishes were washed three times for 5 min, the primary antibodies were detected by further incubation with anti-rabbit, anti-mouse or anti-goat Alexa Fluor 546 or Alexa Fluor 488 secondary antibodies (Beyotime, China). For F-actin staining, the cells were incubated with a 50 μg/ml fluorescent phalloidin conjugate solution (Beyotime, China) in PBS for 1 h at room temperature. VECTASHIELD Fluorescent Mounting Medium with DAPI (VECTOR LABORATORIES, USA) was applied to prevent fluorescence quenching and for nucleus identification. Cell images were captured using a Leica fluorescence microscope (Wetzlar, Germany) with an oil immersion lens and a DC100 digital camera.

### Cell proliferation analysis

Cells were seeded in 96-well plates (5000 cells per well) overnight before transfection, and cell proliferation assays were conducted using the Cell-Light™ 5-ethynyl-2′-deoxyuridine imaging detection kit according to the manufacturer's instructions (RiboBio, China). The measurements were performed in triplicate.

### Statistical analysis

The statistical analyses were performed using the SPSS 15.0 statistical software (SPSS Inc., USA). Comparisons between two groups were conducted with Student's t-test, and correlation analyses were subjected to the Pearson or Spearman correlation test. The OS was calculated using the Kaplan–Meier method, and significant differences were compared with the log-rank test. Fisher's exact test was applied to assess the relationship between clinicopathologic variables and JWA or HER2 expression. Furthermore, univariate and multivariate analyses using Cox regression models were utilized to assess the prognostic value of JWA and HER2. All the P values were two-sided, and P values <0.05 were considered statistically significant.

## SUPPLEMENTARY FIGURES AND TABLES


