# Supplementary material for: JWA loss promotes cell migration and cytoskeletal rearrangement by affecting HER2 expression and identifies a high-risk subgroup of HER2-positive gastric carcinoma patients
Source: Oncotarget. 2016 May 6;7(24):36865–84. doi: 10.18632/oncotarget.9211 (PMC5095045; doi:10.18632/oncotarget.9211)
Supplement: Supplementary file 1 [file oncotarget-07-36865-s001.pdf]

SUPPLEMENTARY FIGURES AND TABLES

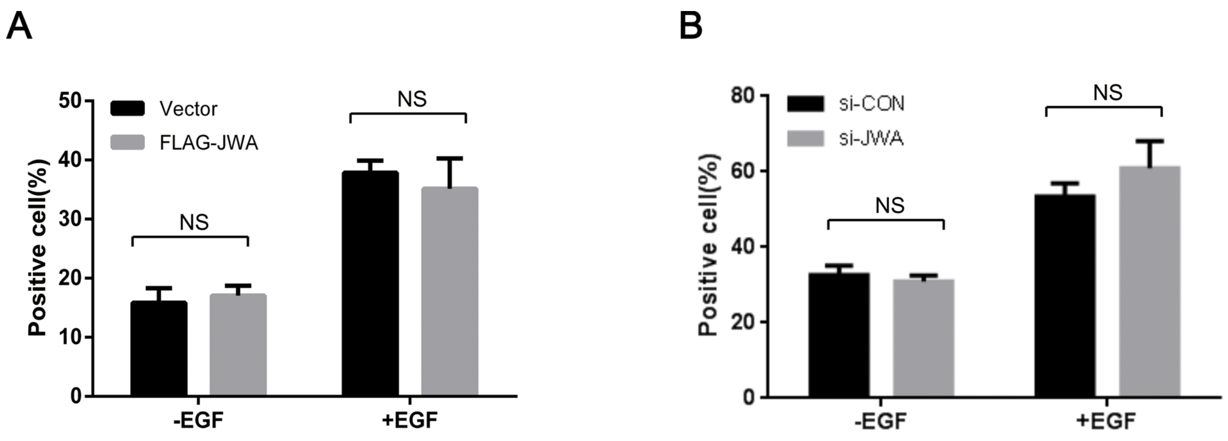

Supplementary Figure S1: The impact of JWA on cell proliferation rate. NCI-N87 and HGC-27 cells were seeded on the 96-well plates before serum-starving for 12 h and transfection with overexpression plasmids and siRNAs for 2 or 3 days, then treated without or with EGF (100 ng/ml) for 20 min. EdU assays was performed using the Cell-Light™ 5-ethynyl-2′-deoxyuridine imaging detection kit according to the manufacturer's instructions. Images were acquired and the fluorescence intensity was quantified under a laser-scanning microscope. The intensity was normalized to blank control and positive percentage was presented in the bar graphs representing the mean and SD of three independent experiments. NS, non-significant, $P>0.05$, 2-sided chi-square test.

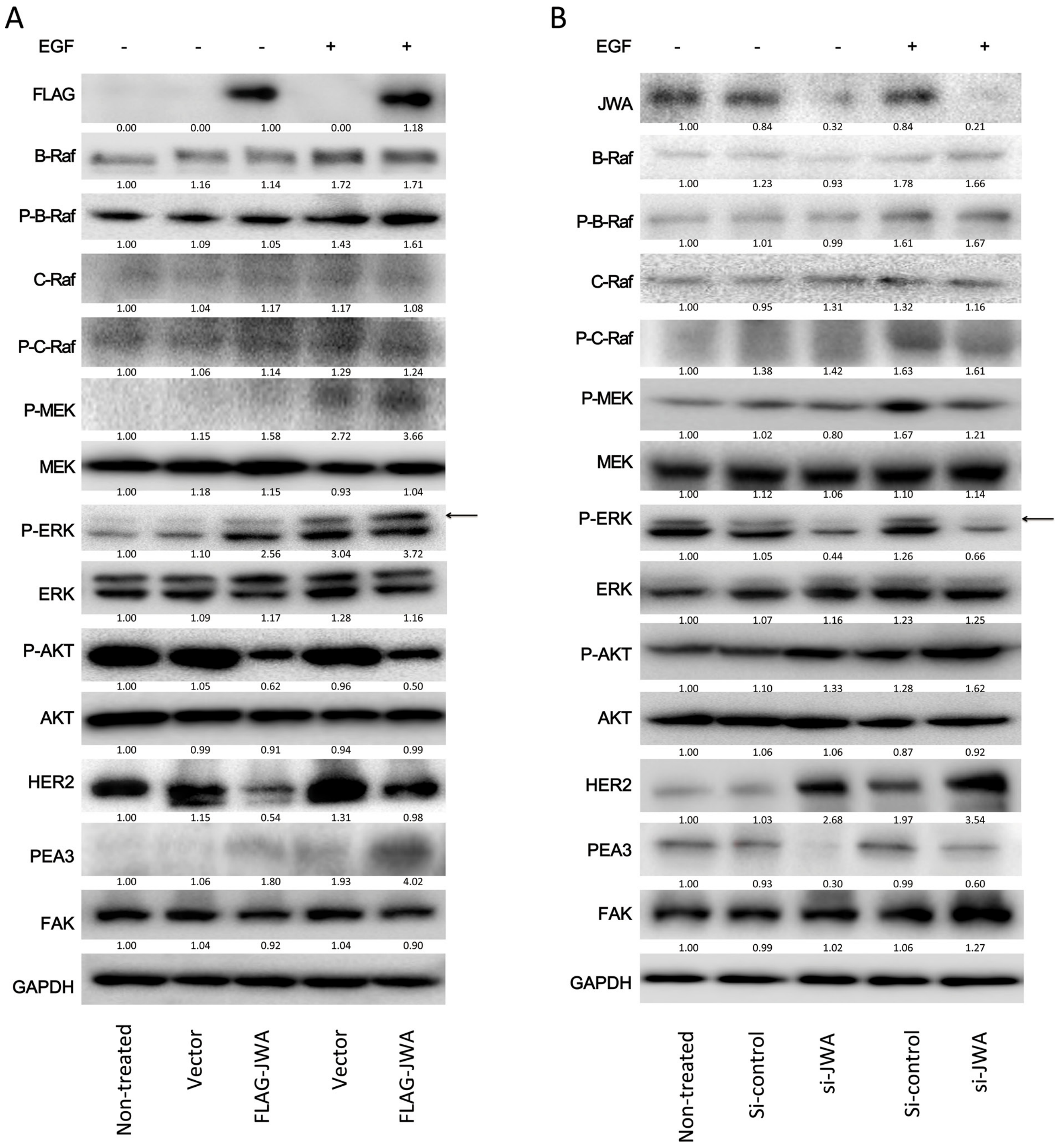

Supplementary Figure S2: Overexpression and silencing of JWA in HGC-27 and NCI-N87 cell lines with untreated control. NCI-N87 and HGC-27 cells transfected with overexpression plasmids and siRNAs were serum-starved for 12 h and then treated without or with EGF (100 ng/ml) for 20 min. FLAG-JWA, JWA, HER2, PEA3, FAK, B-raf, p-B-raf, c-raf, p-c-raf, p-MEK, MEK, p-ERK, ERK, p-AKT, AKT were examined by western blot analysis in JWA-overexpressing NCI-N87 cells **A.** and JWA-silencing HGC27 cells **B.** Arrows indicate the interested band.

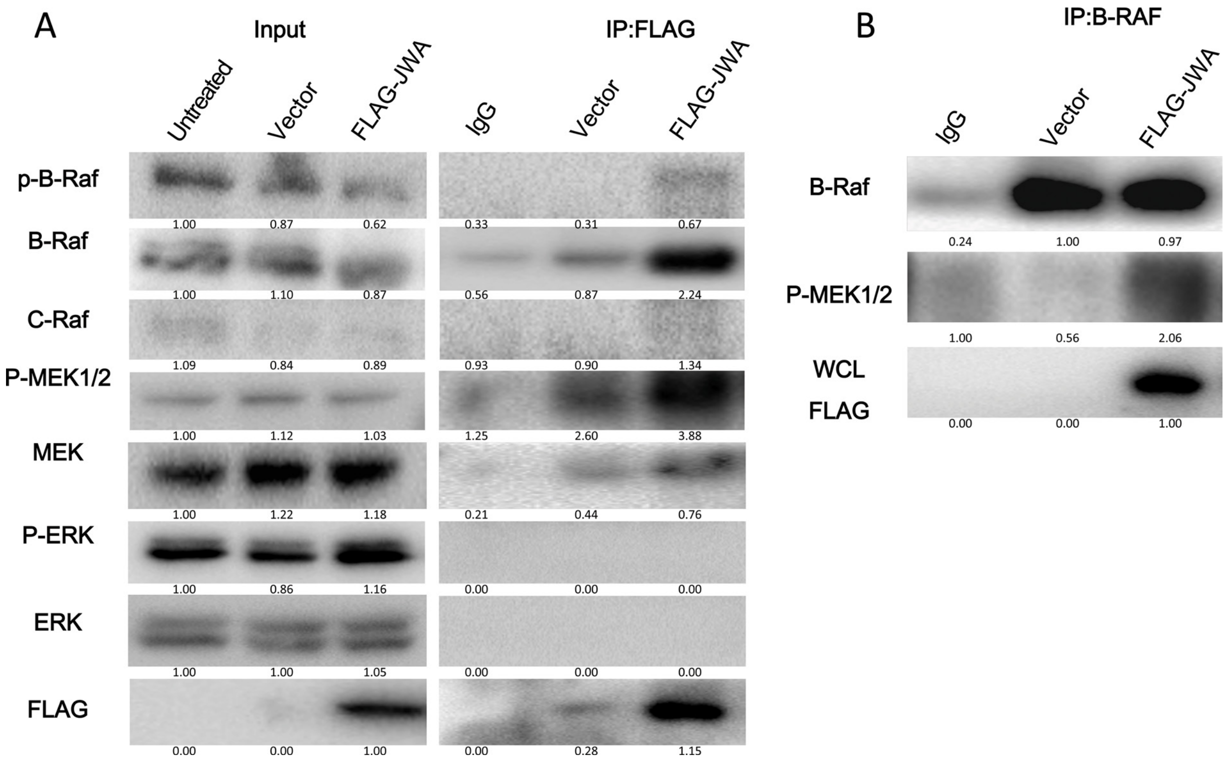

Supplementary Figure S3: The interaction of JWA with MEK/ERK pathway in HER2-negative BGC823 cells. After Flag-JWA was transfected into HGC-27 cells for 48 h, immunoprecipitation assays were conducted. Cells were lysed in 20 mM Tris (pH 8.0), 137 mM NaCl, 10% glycerol, and 0.2% Triton X-100. Anti-anti-Flag **A.** and anti-B-Raf **B.** monoclonal antibodies were incubated with protein G/A-Sepharose beads (Santa Cruz, Santa Cruz, US) for 2 h at 4°C, washed, and incubated for 3 h at 4°C with equal amounts of pre-washed protein lysate. Mouse or rabbit serum IgG (Sigma, St. Louis, MO) were used as controls. Samples were subjected to SDS/PAGE and probed with anti-FLAG-JWA, JWA, HER2, PEA3, FAK, B-raf, p-B-raf, c-raf, p-c-raf, p-MEK, MEK, p-ERK, ERK antibodies.

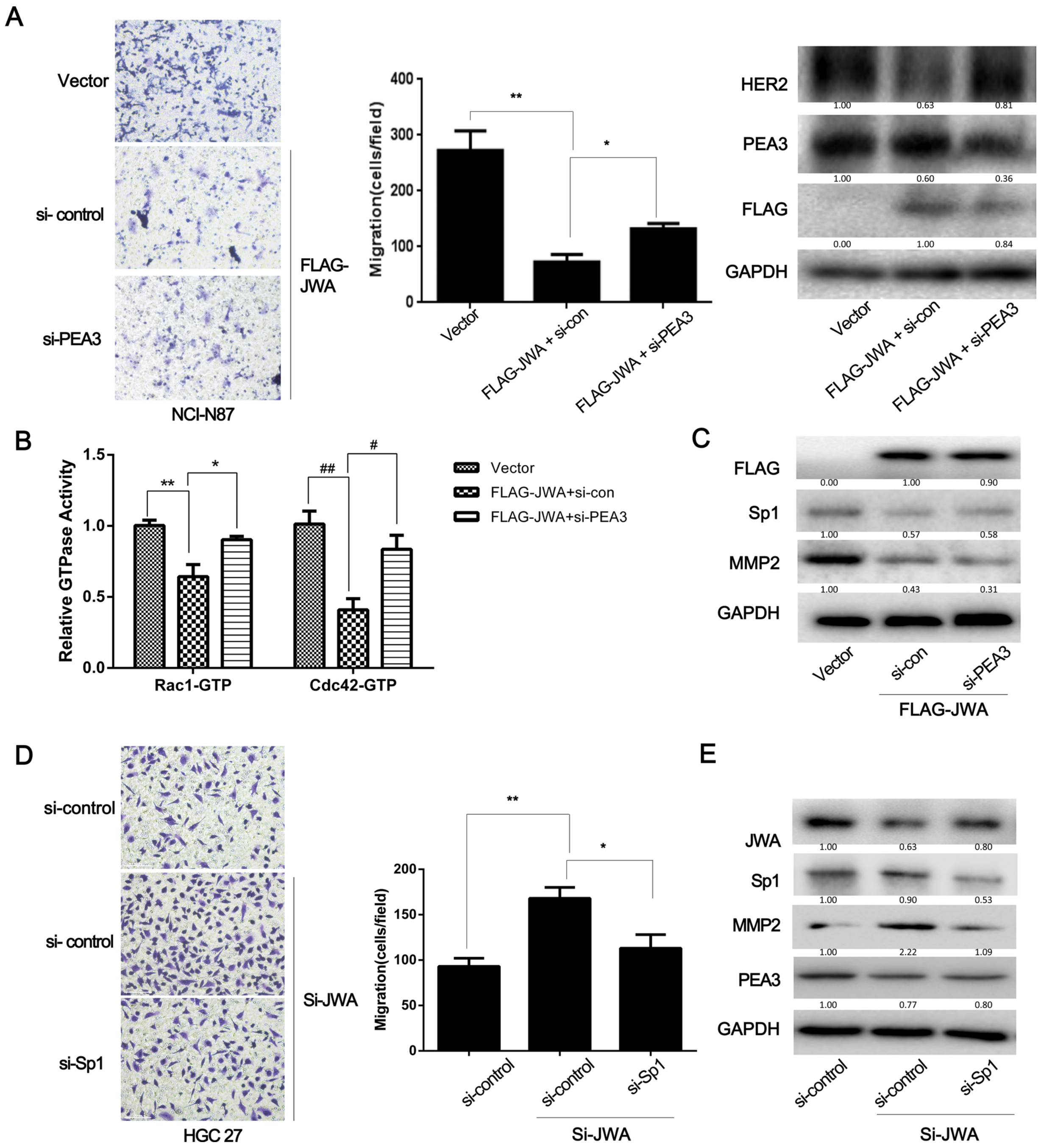

Supplementary Figure S4: The relationship between Sp1- and PEA3-related pathways modulated by JWA. A, B, C. After siPEA3 was transfected into JWA-overexpressing NCI-N87 cells, migration assays were conducted (A, Left panel), and the results are presented in the bar diagram (the numbers of cells on the lower surface of the transwells per field at 200x magnification in five random fields) (A, Middle panel, * $P<0.05$ and ** $P< 0.01$; Student's t-test). The cell lysates were then subjected to immunoblotting to detect FLAG-JWA, PEA3 and HER2 (A, Right panel), Sp1 and MMP2 (C) as well as to G-LISA assays (B) to assess Rac1 and Cdc42 Rho GTPase activity. The data are presented as the mean ± SD of three experiments. * $P<0.05$ and ** $P<0.01$ compared with vector Rac1 activity; # $P<0.05$ and ## $P<0.01$ compared to vector Cdc42 activity; Student's t-test. **D, E.** si-Sp1 RNA was transfected into JWA-deficient HGC-27 cells. Then migration assays were done (D, Left panel), and the results are presented in the bar diagram (the numbers of cells on the lower surface of the transwells per field at 200x magnification in five random fields) (D, Middle panel, * $P<0.05$ and ** $P< 0.01$; Student's t-test). Cell lysates were analyzed by Western blot using anti-JWA, -PEA3, -Sp1 -MMP2 and -GAPDH antibodies (E).

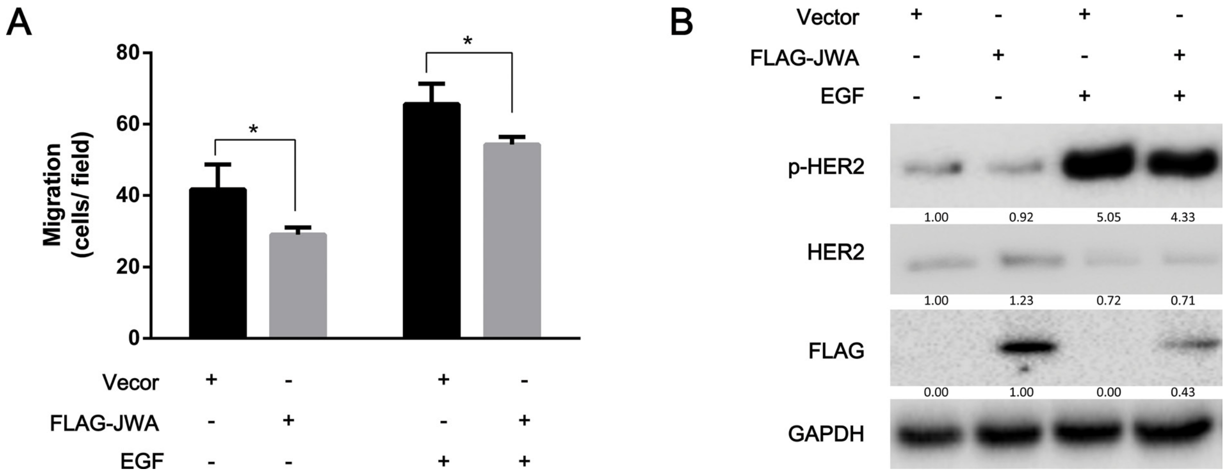

Supplementary Figure S5: The involvement of JWA in cell motility and HER2 expression in HER2-negative BGC-823 cells. BGC823 cells overexpressing JWA or the vector (A) were seeded into the upper compartments of transwells in the absence or presence of 100 ng/ml EGF for 24 h after a 12-h serum starvation. **A.** Migration assays were conducted, and the results are presented in the bar diagram (the numbers of cells on the lower surface of the transwells per field at 200x magnification in five random fields) (* $P<0.05$; Student's t-test). Error bars indicate the S.E.M. of three independent experiments in triplicate. **B.** The cell lysates were then subjected to western blot to detect FLAG, p-HER2 and HER2. GAPDH was used as the loading control.

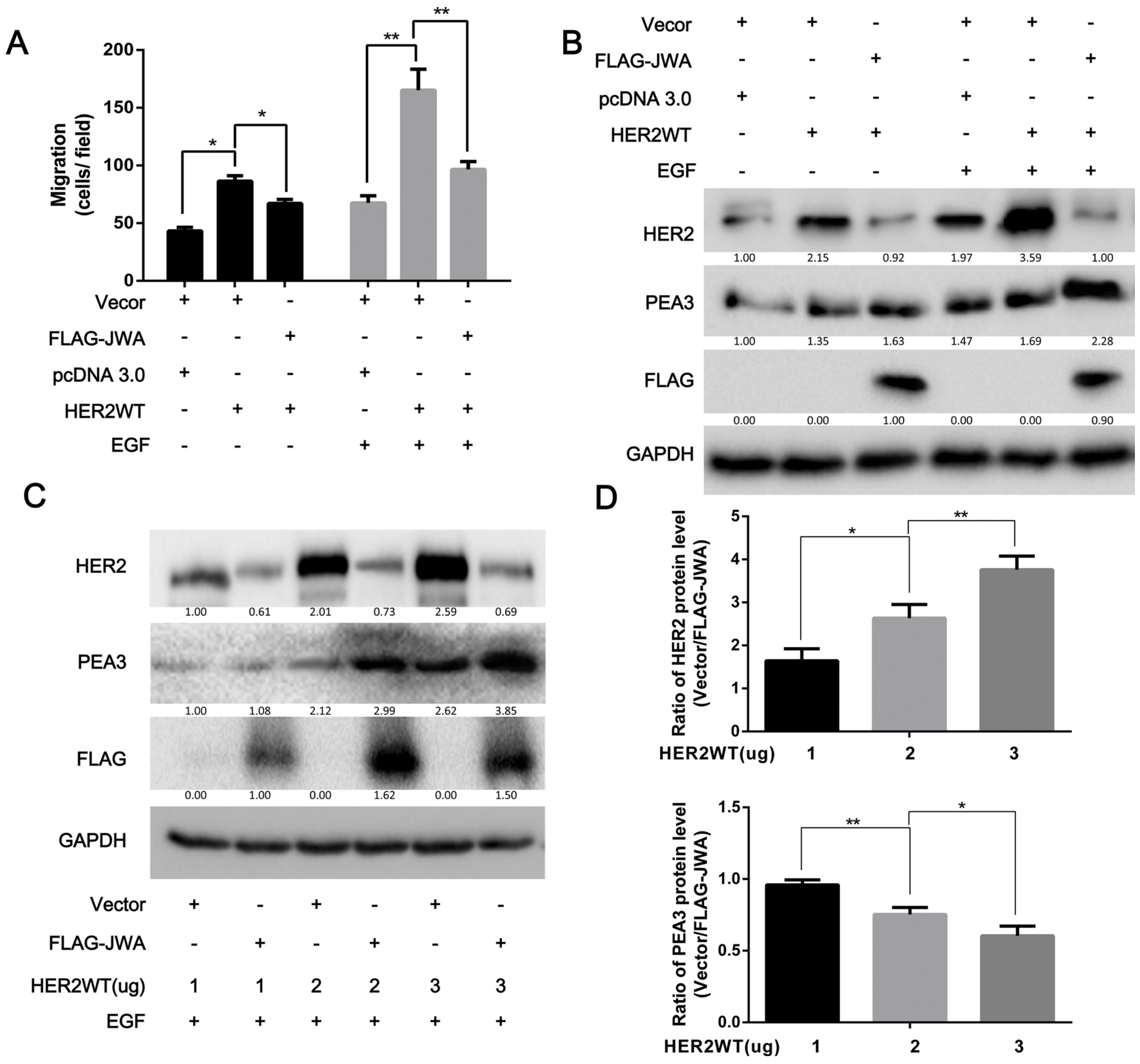

Supplementary Figure S6: JWA correlates with cell motility and HER2 expression in JWA-HER2-co-expressed BGC-823 cells. BGC823 cells were co-infected with 3ug **A, B.** or 1-3ug **C, D.** HER2WT plasmids and 3ug FLAG-JWA or its corresponding vector plasmids. Then, Chamber assays were conducted in the absence or presence of 100 ng/ml EGF for 24h after a 12-h serum starvation. (A) The number of cells on the lower surface of the transwells was counted in five random fields. Error bars indicate the S.E.M. of three independent experiments in triplicate. Two-tailed Student's t-test; *$P < 0.05$, ** $P < 0.01$. (B, C) The expression of FLAG, HER2 and PEA3 were determined by western blot. (D) The intensity of the PEA3 and HER2 protein bands were analyzed by densitometry and normalized to GAPDH. The grey value ratios of Vector to FLAG-JWA in respect to HER2 (Upper panel) or PEA3 (Lower panel) protein level were plotted as the mean ± standard deviation (SD) of 3 independent replicates with the increasing amount of HER2WT plasmids. * $P < 0.05$; ** $P < 0.01$ two-tailed Student's t-test.

Supplementary Table S1: Primer sequences for real-time RT-PCR

Gene	Primer sequences
HER2/neu	F:5'-TGTGACTGCCTGTCCCTACAA-3'
	R: 5'-CCAGACCATAGCACACTCGG-3'
JWA	F:5'-GGAGGAGTCATTGTGGTGC-3'
	R: 5'-GAAGTCTCAGGGATGCGTG-3'
PEA3	F:5'GATGAAAGCCGGATACTTGGAC-3'
	R: 5'-TTCGCGCAAGCTCCCATTT-3'
YB-1	F:5’-TAGACGCTATCCACGTCGTAG-3’
	R:5’-ATCCCTCGTTCTTTTCCCCAC-3’
EGR2	F:5’-TCAACATTGACATGACTGGAGAG-3’
	R:5’-AGTGAAGGTCTGGTTTCTAGGT-3’
YY1	F:5'-ACGGCTTCGAGGATCAGATTC-3'
	R:5'-TGACCAGCGTTTGTTCAATGT-3'
AP-2	F:5'-AGGTCAATCTCCCTACACGAG
	R:5'-GGAGTAAGGATCTTGCGACTGG
FOXP3	F:5’-TCCGTCCTGCAAGGATATTGT-3’
	R:5’-ATGGGGTGCTCTTAATTGTTGAT-3’
GAPDH	F:5'-GCCGGTGCTGAGTATGTC-3'
	R:5'-CTTCTGGGTGGCAGTGAT-3'

Supplementary Table S2: The following antibodies used in the study

Antibodies	Host Species	Clonality	Dilution	Brand	Application
EGFR	rabbit	monoclonal	1:1000	Epitomics, China	WB
p-Tyr1068EGFR	rabbit	polyclonal	1:1000	Abcam, UK	WB
HER2	rabbit	polyclonal	1:1000	Santa Cruz, US	WB
	rabbit	monoclonal	1:200	Epitomics, China	IHC
p-Tyr1248HER2	rabbit	polyclonal	1:1000	Signalway Antibody, US	WB
HER3	rabbit	monoclonal	1:1000	Epitomics, China	WB
p-Tyr1328Her3	rabbit	polyclonal	1:1000	Signalway Antibody, US	WB
AKT	rabbit	monoclonal	1:1000	Epitomics, China	WB
pS473AKT	rabbit	polyclonal	1:1000	Signalway Antibody, US	WB
JNK	rabbit	monoclonal	1:1000	Cell Signaling Technology, US	WB
P-JNK	rabbit	monoclonal	1:1000	Cell Signaling Technology, US	WB
ERK	rabbit	monoclonal	1:1000	Epitomics, China	WB
P-ERK	rabbit	monoclonal	1:1000	Epitomics, China	WB
p-PAK1/2	rabbit	monoclonal	1:1000	Epitomics, China	WB
PAK1/2	rabbit	polyclonal	1:1000	Signal way Antibody, US	WB
PEA3	rabbit	polyoclonal	1:400	Abcam, UK	WB
Beta-Tubulin	rabbit	monoclonal	1:1000	Beyotime, China	WB
GAPDH	mouse	monoclonal	1:1000	Beyotime, China	WB
Actin	mouse	monoclonal	1:1000	Beyotime, China	WB
Histone H3	rabbit	monoclonal	1:1000	Beyotime, China	WB
FLAG-tag	mouse	monoclonal	1:2000	MBL, Japan	WB
	mouse	polyclonal	1:400	Beyotime, China	IF
JWA	mouse	polyclonal	1:200	produced in our laboratory	WB
	goat	polyclonal	1:50	Abcam, UK	IHC
	goat	polyclonal	1:200	Novus Biologicals, US	IF

WB, Western Blotting; IHC, Immunohistochemistry; IF, Immunofluorescence.
